# Mobility, risk behavior and HIV/STI rates among female sex workers in Kaiyuan City, Yunnan Province, China

**DOI:** 10.1186/1471-2334-10-198

**Published:** 2010-07-09

**Authors:** Haibo Wang, Ray Y Chen, Gerald B Sharp, Katherine Brown, Kumi Smith, Guowei Ding, Xia Jin, Junjie Xu, Ruiling Dong, Ning Wang

**Affiliations:** 1 Chinese Center for Disease Control and Prevention, 27 Nanwei Rd, Beijing 100050, China; 2 National Institute of Allergy and Infectious Diseases, National Institutes of Health, 6700B Rockledge Drive, Bethesda, MD, USA; 3 National Center for AIDS/STD Prevention and Control, Chinese Center for Disease Control and Prevention, 27 Nanwei Rd, Beijing 100050, China; 4 Key Laboratory of Immunology of AIDS, Ministry of Health, First Affiliated Hospital, China Medical University, 155 Nanjingbei Rd, Shenyang 110001, China; 5 Shenzhen International Travel Health Care Center, Shenzhen Port Hospital, ShengHuo District HuangGang Port, ShenZhen 518033, China

## Abstract

**Background:**

The mobility of female sex workers (FSWs) is a factor in the geographic spread of human immunodeficiency virus (HIV) and other sexually transmitted infections (STIs). This study describes FSW mobility patterns in a high risk area of China to identify factors associated with increased mobility, and to study the incidence and prevalence of HIV/STIs in this group.

**Methods:**

270 FSWs recruited from a baseline cross-sectional study were invited to participate in a one-year monthly follow-up cohort study in Kaiyuan City, Yunnan Province, China from 2006 to 2007. Laboratory tests were conducted for HIV/STIs at baseline, 6 and 12 months.

**Results:**

A total of 117 (43.3%) FSWs moved to another city during the year. Risk factors for increased mobility included being from another city within Yunnan (adjusted hazard ratio [AHR] 1.67, 95% confidence interval [CI] 1.09-2.56), being from outside Yunnan (AHR 1.58, 95% CI 1.04-2.54), and working in lower risk entertainment establishments (AHR 1.55, 95% CI 1.03-2.35). HIV-positive subjects, drug users and FSWs in higher risk venue were less likely to change residence, less likely to use condoms with clients, and earned less per client, but had more working locations and more clients each month.

**Conclusions:**

The least mobile FSWs were from Kaiyuan, worked in higher risk venues, were more likely to use drugs and be HIV-infected. Because FSWs characteristics differ according to the venue at which they work, future prevention work should tailor programs according to venue with a particular focus on FSWs in higher risk venues.

## Background

Since the 1980 s, commercial sex work has become increasingly prevalent in China. The Chinese Public Security office estimated that there were 4 to 6 million sex workers nationwide in 2000, a 160-fold increase since 1985 [1]. Female sex workers (FSWs) in developing countries often relocate to places with greater numbers of potential clients, such as military camps, mining towns and roadside truck stops [2,3]. Such mobility can facilitate the spread of the human immunodeficiency virus (HIV) and sexually transmitted infections (STIs) from higher prevalence areas to lower prevalence areas.

In China, HIV infection rates are highest in Yunnan Province [4]. Although the HIV epidemic there has been driven predominantly by injection drug use, sexual transmission has been playing an increasingly greater role in recent years [5,6]. Research into FSW mobility is thus particularly important because of its role in facilitating the transmission of HIV/STIs.

Kaiyuan City is located in southern Yunnan Province, and lies along drug trafficking routes brining heroin into China through Myanmar and Vietnam. Prevalent injection drug use has spurred the spread of HIV in Yunnan province, leading to infection rates of epidemic proportions; HIV prevalence among FSWs has reached 10.3% in some locations [6]. Because of the high mobility of FSWs, there is considerable risk for the transmission of HIV from this high-risk area of Kaiyuan to other currently lower risk areas. The primary objective of this study was to describe the mobility patterns of FSWs in this high risk area of China, to identify factors associated with increased mobility, and to study the incidence and prevalence of HIV/STIs in this group.

## Methods

This study received the approval from both the national and local Yunnan institutional review boards, and was conducted by the Chinese Center for Disease Control and Prevention (China CDC) in Beijing in cooperation with provincial and local CDC staff in Yunnan. A baseline cross-sectional survey was conducted of all available FSWs in the study area. Local CDC outreach workers publicized the study to all entertainment venues and bosses and invited women working as sex workers to participate. The inclusion criteria were women aged ≥16 years, self-reported to have sold sex for money within the previous 3 months, and who agreed to testing and counseling for HIV/STIs. Additional details of the recruitment have been previously published [6]. After the baseline survey, all HIV-positive and all drug-using subjects who returned for post-test counseling were invited to participate in a one-year monthly follow-up cohort study. A systematic sampling system was used to select HIV-negative, non drug-using individuals for participation. Within each income stratum of <$7, $7-$14 and >$14 per sex service, participants were assigned a number according to the order in which they attended post test-counseling. Even or odd numbered participants within each income strata were selected for participation upon the random selection of 1 (designating odd numbered participants would be invited to participate) or 2 (designating even numbered participants would be invited to participate). To be eligible, the designated participants had to provided: 1) written informed consent; 2) their cell phone number or other contact information; 3) information about one or more proxy representatives (friend, peer leader, madam, etc.) who could be contacted in case a participant could not be located; and 4) agreement to be contacted monthly for 12 months to assess current sex venue, reasons for any changes in venues, and sexual behavior with clients and regular sexual partners. Enrolled FSWs were then interviewed monthly either in person at the study center or by cell phone. If study staff were unable to reach the participant directly, the participant's proxy was contacted to obtain follow-up information. Participants were given $3 at each follow-up visit to compensate them for their time and travel.

At baseline, 6 and 12 months, laboratory tests were conducted for HIV, gonorrhea, chlamydia, syphilis, HSV, trichomonas, and urine drug screening with post-test counseling offered one month later. Blood specimens were collected and tested for: 1) herpes simplex virus type 2 (HSV-2) antibody (HerpeSelect-2 ELISA IgG, Focus, USA); 2) syphilis (rapid plasma reagin (RPR) test, Xinjiang Xindi company, China) with confirmation of positive tests by the *Treponema pallidum *particle assay (TPPA) test (Serodia-P·PA-Fujirebio, Fuji, Japan), and 3) HIV-1 antibody (enzyme-linked immunosorbent assay (ELISA), Vironostika HIV Uni-Form plus O, bioMerieux, Holland) with confirmation by western blot (Diagnostics HIV Blot 2.2, Genelabs, USA). Endocervical swabs were collected and tested for *Neisseria gonorrhoeae *and *Chlamydia trachomatis *by polymerase chain reaction (PCR, AMPLICOR, Roche, USA). A wet mount from vaginal swabs was prepared to detect *Trichomonas vaginalis*. Finally, urine was collected for opiate screening (MOP One Step Opiate Test Device, ACON Laboratories, Inc., USA). Participants were classified as using illegal drugs if they either self-reported or tested urine positive for opiates. All subjects were scheduled for a follow-up visit 4-6 weeks later for post-test counseling. All FSWs found to be infected with an STI were referred to the local CDC STI clinic for medical evaluation and treatment, and provided with a 60% discount coupon for STI treatment. Treatment was not provided by the study.

An incident STI infection was defined as testing negative at one visit and subsequently testing positive at the next. Tests that were positive on consecutive visits were considered persistent/recurrent because we did not have STI treatment data to distinguish the two. Syphilis cure was defined as a four-fold decrease in RPR titer after at least 6 months. Subjects without a four-fold decrease were considered persistent/recurrent.

Statistical tests were performed using SAS™ 9.1 software (SAS Institute Inc, Cary, NC, USA). The Cox proportional hazard model was used to identify factors associated with FSW mobility. FSW mobility was defined as moving away from Kaiyuan City during the one year follow-up. Participants lost to follow-up for six months or longer were considered to have moved away from Kaiyuan after their last follow-up visit because outreach workers, familiar with these FSWs and their entertainment venues and bosses, were able to locate most FSWs if they had not moved away. Participants who remained in Kaiyuan but moved to a new living location were categorized as "changed residence." Subjects who were sent to detoxification centers, or withdrew from the study during the 12 months were censored on the date of their last contact. T-tests, Fisher Exact tests, and chi-square tests were used to compare HIV-positive and -negative subjects, as well as illegal drug users and non-drug users. Higher risk venues were defined as locations where FSWs generally charged less than $14 for sexual services, including beauty salons, temporary sublets, and public places (the street or plazas); these FSWs attracted poorer and less educated clients potentially at higher risk of HIV infection, such as drug users. Lower risk venues were defined as locations where FSWs generally charged $14 or more for sex, including karaoke clubs, night clubs, saunas, and hotels; these FSWs attracted a wealthier, better educated clientele [6,7].

## Results

### Baseline characteristics of the study population

Among the 737 FSWs who participated in the baseline cross-sectional survey, 270 (37%) were recruited into the cohort study, including 27 HIV-positive drug users, 20 HIV-positive non-drug users, 36 HIV-negative drug users, and 187 HIV-negative non-drug users. Median age was 24 years (interquartile range [IQR], 20-29 years), 70% were of Han ethnicity, and over 80% had registered permanent residences in Yunnan. As shown in Table 1, women recruited into the follow-up study were older, more likely to use drugs, and more likely to be HIV positive compared to women who did not enter the follow-up study.

**Table 1:** Demographic characteristics of female sex workers in the follow-up cohort study.

Demographics		Recruited in the cohort study		*P *value
			
		Yes (N = 270)	No (N = 467)	
Age (years)	16-20	76 (28.2)	149 (31.9)	0.0388
	21-25	84 (31.1)	171 (36.6)	
	26-52	110 (40.7)	147 (31.5)	
Nationality	Han	190 (70.4)	302 (64.7)	0.1134
	Other	80 (29.6)	165 (35.3)	
Registered permanent residence	Kaiyuan city	123 (45.6)	174 (37.3)	0.0826
	Other cities in Yunnan	100 (37.0)	195 (41.7)	
	Outside Yunnan	47 (17.4)	98 (21.0)	
Education level	< 9 years	133 (49.3)	254 (54.4)	0.1790
	≥9 years	137 (50.7)	213 (45.6)	
Marital status	Married or cohabitation	85 (31.5)	140 (30.0)	0.6695
	Single, separated, divorced, or widowed	185 (68.5)	327 (70.0)	
Earnings per sex	≤$7	44 (16.3)	65 (13.9)	0.6586
	$7~$14	128 (47.4)	232 (49.7)	
	> $14	98 (36.3)	170 (36.4)	
Entertainment venue	Lower risk	163 (60.4)	295 (63.2)	0.4504
	Higher risk	107 (39.6)	172 (36.8)	
Drug use (ever)*	Non-drug user	207 (76.7)	410 (87.8)	< .0001
	Non-injection illegal drug user	22 (8.2)	29 (6.2)	
	Injection drug user	41 (15.2)	28 (6.0)	
HIV*	Negative	223 (82.6)	438 (93.8)	< .0001
	Positive	47 (17.4)	29 (6.2)	
Syphilis	Negative	246 (91.1)	436 (93.4)	0.2626
	Positive	24 (8.9)	31 (6.6)	
*N. gonorrhoeae*	Negative	247 (91.5)	429 (91.9)	0.8563
	Positive	23 (8.5)	38 (8.1)	
*C. trachomatis*	Negative	206 (76.3)	340 (72.8)	0.2973
	Positive	64 (23.7)	127 (27.2)	
*T. vaginalis*	Negative	241 (89.3)	418 (89.5)	0.9159
	Positive	29 (10.7)	49 (10.5)	
HSV-2	Negative	75 (27.8)	160 (34.3)	0.0688
	Positive	195 (72.2)	307 (65.7)	

*All self-reported drug users, urine drug positive subjects, or HIV positive subjects from the baseline survey were recruited into the cohort study.

### Mobility of FSWs

**Figure 1:**
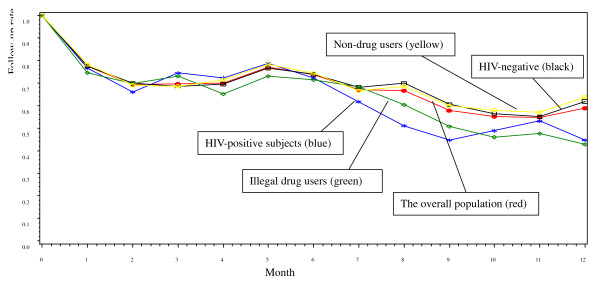
**Overall follow-up rate by month and stratified by HIV sero-positivity and drug using for the 270 female sex workers included in the follow-up study**. The follow-up rate of HIV-positive subjects was significant lower than that of HIV-negative subjects (*P *= 0.045), and the follow-up rate of illegal drug users was significant lower than that of non-drug users (*P *= 0.012).

Figure 1 illustrates the follow-up retention rates for the entire cohort, stratified by subgroups. Overall, 159 (59%) of the 270 subjects enrolled completed the final follow-up visit. HIV-negative subjects (138 [62%]) were significantly more likely to be retained in the study than HIV-positive subjects (21 [45%], *P *= 0.04). FSWs who did not use drugs (132 [64%]) were also significantly more likely to be retained than drug users (27 [43%], *P *= 0.01). Twenty-two drug users, of whom 12 were HIV-positive, were arrested and sent to detoxification centers during the study.

At various points during the one year follow-up, 117 (43.3%) FSWs moved to other cities. Table 2 describes the baseline characteristics of the FSWs stratified by whether or not they moved during the study. Subjects were significantly more likely to move if they were from other cities within Yunnan province (adjusted hazard ratio [AHR] 1.67, 95% confidence interval [CI] 1.09-2.56) or from outside Yunnan (AHR 1.58, 95% CI 1.04-2.54). FSWs who worked in lower risk sex venues were also more likely to move during the year (AHR 1.55, 95% CI 1.03-2.35), compared to those working in higher risk venues.

**Table 2:** Baseline characteristics of the 270 female sex workers recruited into the follow-up cohort study, stratified by whether or not they moved to a different city during the study.

	Characteristic	Cohort members who moved (N = 117) Number (%)	Cohort members who did not move (N = 153) Number (%)	Mobility incidence (/100 person months)	Univariate analysis Hazard Risk (95% Confidence Interval)	Multivariate analysis Hazard Risk (95% Confidence Interval)
Age (years)	16-20	35 (29.9)	41 (26.8)	5.75	1.0	
	21-25	42 (35.9)	42 (27.4)	6.00	1.04 (0.66-1.63)	0.95 (0.61-1.50)
	26-52	40 (34.2)	70 (45.8)	4.13	0.72 (0.46-1.13)	0.63 (0.38-1.04)
Nationality	Han	83 (70.9)	107 (69.9)	5.17	1.0	
	Other	34 (29.1)	46 (30.1)	5.06	0.98 (0.66-1.46)	
Registered	Kaiyuan city	39 (33.3)	84 (54.9)	3.67	1.0	
permanent	Other cities in Yunnan	53 (45.3)	47 (30.7)	6.47	1.74 (1.15-2.63)	1.67 (1.09-2.56)
residence	Outside Yunnan	25 (21.4)	22 (14.4)	6.33	1.70 (1.03-2.81)	1.58 (1.04-2.54)
Education level	< 9 years	55 (47.0)	78 (51.0)	4.84	1.0	
	≥9 years	62 (53.0)	75 (49.0)	5.44	1.13 (0.78-1.62)	
Marital status	Married or cohabitation	37 (31.6)	48 (31.4)	5.14	1.0	
	Single, separated, divorced, or widowed	80 (68.4)	105 (68.6)	5.13	0.99 (0.67-1.46)	
Earnings per sex	≤$7	14 (12.0)	30 (19.6)	3.77	1.0	
	$7~$14	49 (41.9)	79 (51.6)	4.36	1.15 (0.64-2.09)	
	> $14	54 (46.1)	44 (28.8)	6.89	1.79 (1.00-3.23)	
Drug use (ever)	Non-drug user	97 (82.9)	110 (71.9)	5.44	1.0	
	Non-injection illegal drug user	11 (9.4)	11 (7.2)	6.20	1.12 (0.60-2.08)	1.34 (0.71-2.54)
	Injection drug user	9 (7.7)	32 (20.9)	2.84	0.52 (0.26-1.02)	0.72 (0.33-1.54)
HIV	Negative	101 (86.3)	122 (79.7)	5.31	1.0	
	Positive	16 (13.7)	31 (20.3)	4.24	0.80 (0.47-1.36)	1.12 (0.63-1.99)
Any STIs	Negative	26 (22.2)	22 (14.4)	6.55	1.0	
	Positive	91 (77.8)	131 (85.6)	4.84	0.73 (0.47-1.13)	
Duration of	< 1 year	31 (26.5)	40 (26.1)	5.16	1.0	
commercial sex	1-2 years	53 (45.3)	59 (38.6)	5.70	1.10 (0.70-1.71)	
Work	≥3 years	33 (28.2)	54 (35.3)	4.42	0.85 (0.52-1.39)	
Entertainment venue	Higher risk	35 (29.9)	72 (47.1)	3.77	1.0	
	Lower risk	82 (70.1)	81 (52.9)	6.07	1.59 (1.07-2.36)	1.55 (1.03-2.35)

(HIV sero-positivity and drug users were added into the multivariate analysis model)

### Changes in FSWs residence, work location, and sexual behaviors

Table 3 shows changes in FSW residence, work location, and behaviors stratified by HIV infection status, drug use and riskiness of venue. HIV-positive subjects were less likely to change residence, earned less per client, had more clients each month, and used condoms more consistently with regular partners. About half of the HIV-positive women changed their residence during the follow-up period, compared to two thirds of HIV-negative subjects. Compared to women who did not use drugs, drug users were less likely to change residence, less likely to use condoms with clients, earned less per client, but had more working locations and more clients each month. The mean number of working locations of drug users and non-drug users were about 1.5 vs. 1.1 each month, respectively (p < 0.01). FSWs in higher risk venue were less likely to change residence, less likely to use condoms with clients, earned less per client, but had more months engaging in sexual service, more working locations and more clients each month.

**Table 3:** Change in residence, working location, and behaviors during the follow-up cohort study among the 270 female sex workers included, stratified by HIV and drug using status.

Variables		HIV positive (N = 47)	HIV negative (N = 223)	*P *value	Drug users (N = 63)	Non-drug users (N = 207)	*P *value	Higher risk venue (N = 107)	Lower risk venue (N = 163)	*P *value
Change residence during follow-up	Yes	22 (46.8)	150 (67.3)	< .01	28 (44.4)	144 (69.6)	< .01	59 (55.1)	113 (69.3)	0.02
	No	25 (53.2)	73 (32.7)		35 (55.6)	63 (30.4)		48 (44.9)	50 (30.7)	
Frequency of changing residence in one year		1.8	2.4	0.18	1.4	2.5	< .01	1.9	2.5	0.09
Months of providing sexual services in one year		10.6	10.7	0.88	11.1	10.6	0.22	11.3	10.3	< .01
Average number of working locations in one month		1.4	1.2	0.11	1.5	1.1	< .01	1.5	1.1	< .01
Average number of clients in one month		17.7	10.7	< .01	21.1	9.1	< .01	19.2	7.1	< .01
Used condoms with every client in previous year	Yes	30 (63.8)	158 (70.8)	0.34	37 (58.7)	151 (72.9)	0.03	59 (55.1)	129 (79.1)	< .01
	No	17 (36.2)	65 (29.2)		26 (41.3)	56 (27.1)		48 (44.9)	34 (20.9)	
Condoms were provided at the working locations	Yes	30 (63.8)	139 (62.3)	0.85	41 (65.1)	128 (61.8)	0.64	61 (57.0)	108 (66.3)	0.12
	No	17 (36.2)	84 (37.7)		22 (34.9)	79 (38.2)		46 (43.0)	55 (33.7)	
Earnings per client		86.7	130.2	< .01	90.5	131.9	< .01	81.6	150.2	< .01
Months of working in two or more locations		4.4	3.8	0.35	5.0	3.6	0.03	4.7	3.4	0.02
Had primary work location	Yes	18 (38.3)	113 (50.7)	0.12	32 (50.8)	99 (47.8)	0.68	57 (53.3)	74 (45.4)	0.21
	No	29 (61.7)	110 (49.3)		31 (49.2)	108 (52.2)		50 (46.7)	89 (54.6)	
Changed primary work location	Yes	4 (22.2)	36 (31.9)	0.58	8 (25.0)	32 (32.3)	0.51	18 (31.6)	22 (29.7)	0.82
	No	14 (77.8)	77 (68.1)		24 (75.0)	67 (67.7)		39 (68.4)	52 (70.3)	
Earnings per month aside from sexual services		270.3	530.4	< .01	328.5	532.8	< .01	265.1	629.5	< .01
Had regular sex partner	Yes	34 (72.3)	156 (67.0)	0.74	42 (66.7)	148 (71.5)	0.46	76 (71.0)	114 (69.9)	0.85
	No	13 (27.7)	67 (30.0)		21 (33.3)	59 (28.5)		31 (29.0)	49 (30.1)	
Months per year of having sex with regular partner		4.7	6.4	0.55	7.8	7.1	0.46	7.7	6.9	0.41
Consistently used condoms with regular partner	Yes	12 (35.3)	26 (16.7)	0.01	8 (19.0)	30 (20.3)	0.86	14 (18.4)	24 (21.1)	0.66
	No	22 (64.7)	130 (83.3)		34 (81.0)	118 (79.7)		62 (81.6)	90 (78.9)	

The reasons for changing residence included attending to family needs (43.2%), to earn more money (16.7%), because business was not good in previous location (9.9%), personal safety (6.8%), bored with one place and willing to change (6.1%), dislike of previous location (4.2%) and other (13.1%). Reasons for changing work location were because business was not good in previous location (35.9%), to earn more money (34.0%), dislike of previous location (7.6%), bored with one place and willing to change (5.0%), personal safety (4.6%), family needs (3.1%), dislike of the boss or madam (3.1%) and other (6.7%).

### Prevalence and incidence of HIV and other STIs

At the baseline cross-sectional survey among 737 FSWs, HIV-1 prevalence was 10.3%, syphilis 7.5%, *N. gonorrhoeae *8.3%, *C. trachomatis *25.9%, *T. vaginalis *10.6%, and HSV-2 68.1%. Of the 270 FSWs recruited into the cohort study, 184 were retested at six months and 157 at twelve months. Table 4 describes the prevalence, incidence, and persistence/recurrence of STIs at each time point among the 270 in the cohort study. HSV-2 had the highest prevalence and incidence, compared to syphilis with the highest persistence/recurrence. At baseline, FSWs from higher risk venues were significantly more likely to have HIV (p < 0.01), syphilis (p < 0.05), and HSV-2 (p < 0.01) but not more likely to have gonorrhea, chlamydia, or trichomonas (p > 0.05).

**Table 4:** Prevalence, incidence and persistence/recurrence of HIV and sexually transmitted infections among the 270 female sex workers included in the study.

	Syphilis	*N. gonorrhoeae*	*C. trachomatis*	*T. vaginalis*	HSV-2
Baseline prevalence (N = 270)	24 (8.9%)	23 (8.5%)	64 (23.7%)	29 (10.7%)	195 (72.2%)
6^th ^month prevalence (N = 184)	17 (9.2%)	12 (6.5%)	35 (19.0%)	20 (10.9%)	136 (73.9%)
12^th ^month prevalence (N = 157)	16 (10.2%)	10 (6.4%)	22 (14.0%)	16 (10.2%)	129 (82.2%)
Incidence (/100 person years)	2 (1.2)	16 (10.2)	34 (26.7)	20 (13.3)	13 (30.4)
Persistence or recurrence per 6-month (%)	29 (93.5%)	5 (20.8%)	22 (33.3%)	16 (40%)	__

Two subjects HIV-seroconverted at the six month survey and a third FSW seroconverted at the twelfth month survey. Of the two FSWs who seroconverted at six months, one was also an IDU. In the first 6 months, before being informed that she had become HIV positive, she had more clients (mean 240/month) than in the subsequent six months (mean 150/month), but she reported consistent condom use with every client both before and after being notified of her HIV status. The second FSW who converted at six months was not a drug user. She solicited clients on the street and her average price per sex act was about 20 RMB or about US$3. She changed her residence for family reasons in the second six-month period. During the first 6 months, she had more clients (mean 23/month) than during the following six months (mean 5/month). She did not use condoms routinely in the first 6 months but reported always using condoms with clients and her regular partner afterwards.

## Discussion

In this study, in which a cohort of 270 FSWs were followed monthly for one year to understand mobility, risk patterns, and HIV/STI rates, we found that, in general, FSWs were highly mobile with 43% of subjects moving between cities over the course of the year. Mobility patterns, however, differed by type of FSW with FSWs not from Kaiyuan and working in lower risk entertainment establishments significantly more likely to move away than those from Kaiyuan and those working in higher risk establishments. Although drug use and HIV status were not correlated with mobility in the overall analysis, the characteristics of drug users and HIV positive women were generally similar to those working in higher risk entertainment establishments. HIV-positiive and drug-using FSWs were less likely to change residences but generally had more working locations. They serviced more clients per month but earned less per client and had less non-sexually related income. Overall HIV/STI rates were high among all FSWs.

Mobility in general and among FSWs in particular has been assumed to contribute to the spread of HIV [8-18]. Our cohort study found that mobility patterns among FSWs in southern China was associated with the type of entertainment establishments in which they worked, finding that FSW in higher risk venues were actually more mobile than those from other establishments. Higher risk venues were defined as locations where FSWs charged less and the FSWs in these locations have many characteristics in common with HIV positive and drug using sex workers. Thus, although FSWs in general are quite mobile, the highest risk FSWs in our study were less mobile than those at lower risk. The underlying motivation for mobility may be related to income as the main reasons for changing residence and working locations were "business not good" and "to earn more money". FSWs whose home town was in other cities were also more likely to move more frequently, most likely because they prefer to engage in sex work outside of their home town to protect their identify and for fear of bringing shame to their family. Those who have moved once also seemed more likely to move again. Why drug users and HIV positive women had a significantly lower follow-up rate (Figure 1) but were not significantly associated with mobility in the multivariate analysis (Table 2) is not clear but may in part be due to the women sent to detention centers. These women disappeared from follow-up but were considered censored rather than moved in the multivariate analysis.

The reasons why higher risk FSWs, who include more drug-using and more HIV-positive women, were less mobile were not fully understood. One reason may be because their lower income means they have fewer resources to move. In addition those with drug addiction may find it difficult to move to an unfamiliar town where they do not have connections to the local illicit drug market. Finally prevention and treatment services for those seeking AIDS treatment or drug treatment can only access such services as methadone or antiretroviral treatment in the location in which they are permanently registered, further restricting their mobility. These hypotheses are points of interest on which further research is recommended.

The study findings have several implications for future interventions targeting FSW at risk of HIV and STD infection. Those working at higher risk venues are a primary target for prevention work because they tend to be less mobile; however, this study also found an overall high mobility and high rates of HIV/STIs among all FSWs, suggesting that all prevention programs addressing sexual transmission will need to consider FSW mobility as a factor in their work.

Considering the significant differences between FSWs working in higher and lower risk venues, interventions should be tailored for each type. FSWs in higher risk venues included more drug users and HIV-positive women who reported lower condom use rates, earned less per client, had worked as a commercial sex worker for longer, and had more clients each month. These FSWs are not only more vulnerable to HIV/STI infection themselves but also place greater numbers of sexual partners at risk of infection once infected. Prevention efforts and program linking these women to treatment should specifically target FSWs working on the street, in temporary sub-lets, and in beauty salons and those targeting FSWs in lower risk venues should account for their higher mobility, providing information about support organizations in other cities.

Our study found very high STIs rates among study subjects. The prevalence of any STI (79%) in the FSWs in our study was similar to that found in another study (84%) of FSWs in Yunnan [19]. In our study, *C. trachomatis *was the most common bacterial STI followed by *T. vaginalis *and *N. gonorrhoeae*, similar to the rates found FSWs in Yunnan (58.6%, 43.2%, and 37.8% respectively) and Guangzhou (32%, 12.5%, and 8%) [16,17]. HIV prevalence in our study (10%) was much higher than other FSW studies conducted in Guangzhou (1.4%) [20], Henan Province, (0.4%) [21] and Sichuan Province (0.6%) [22]. These differences, may be due to regional and temporal differences. Yunnan has the highest prevalence of HIV in China, largely due to IDU [23].

Our results also demonstrated a high incidence and persistence/recurrence of STIs, demonstrating high risk sexual behavior and poor treatment seeking behavior with the possibly substandard treatment of infections. In particular, syphilis had the highest persistence or recurrence (93.5%). At baseline, significantly more FSWs infected with syphilis were located at higher risk entertainment venues, in contrast to those infected with gonorrhea, chlamydia, and trichomonas. Because FSWs at higher risk entertainment venues earned less yet had more sexual exposure (Table 3), it is possible that they were either unable to afford STI treatment or had more recurrence. The role of STIs in HIV transmission has been well reviewed [20,24-27]. Despite suppressive therapy of HSV-2 with acyclovir being shown to reduce genital ulcers and plasma HIV RNA levels in dually infected women [28], HIV transmissions were not reduced [29].

Our study has several limitations. First, participants lost to follow-up for six months or longer were assumed to have moved away. This may have overestimated the FSW mobility as some FSWs lost to follow-up may still live in Kaiyuan City. However, our assumption that the participant moved away was based on outreach efforts by the local CDC outreach workers, who were very familiar with the FSWs. In addition, we also collected contact information from one or more proxy representatives, such as friends or their entertainment venue bosses, who often know the whereabouts of the participants. Through these efforts, we believe that this overestimation is small. Second, to learn more information about risk behavior and mobility pattern of HIV-positive subjects and drug users, we recruited all the HIV-positive subjects and drug users who were willing to participate in our study. Consequently, our study cohort over-represented HIV-positive FSWs and drug users. Third, we were not able to collect reliable STI treatment information, thus were not able to differentiate STI persistence from recurrence. However, whether it was persistence or recurrence does not change the fact that these FSWs continued to test positive and could potentially continue to infect their sexual partners. Barriers to treatment need to be explored to make appropriate treatment more available and accessible. Finally, our study included a relatively small number of FSWs from one location in China. How representative our study is of FSWs in China or elsewhere internationally is not known and will depend on the results of additional studies.

## Conclusions

Our study confirmed the high rates of mobility among FSWs identified by other studies but went on further to demonstrate that FSWs from outside of Kaiyuan and those working in lower risk entertainment venues were significantly more mobile. Those working in higher risk entertainment venues, who tended to be more drug-using and HIV-positive sex workers, were less mobile. The reasons for mobility were most commonly related to increasing income. We furthermore documented high rates of HIV/STIs in this cohort which, when combined with the high rates of mobility, implies that HIV may spread to low-risk areas through mobile FSWs. Future interventions for FSWs can be tailored for high and low risk according to their differing risk factors. Further research is needed to examine the specific reasons for mobility and STI treatment seeking behaviors in order to better inform efforts to reduce HIV/STI risk in this highly vulnerable population.

## Competing interests

The authors declare that they have no competing interests.

## Funding

This study was supported by the Comprehensive International Program of Research on AIDS (CIPRA) grant from the National Institute of Allergy and Infectious Diseases, U.S. National Institutes of Health (U19 AI51915-05).

## Authors' contributions

NW was the PI for the study, HW was the lead author for the paper; NW, HW, RYC, GBS, GD, and JX contributed the design of the study; HW performed all the statistical analyses; NW, HW, RYC, GBS, KB, KS, GD, XJ, JX, and RD oversaw data collection at the study site, and all the authors contributed to the write up. All authors read and approved the final manuscript.

## Pre-publication history

The pre-publication history for this paper can be accessed here:

http://www.biomedcentral.com/1471-2334/10/198/prepub
